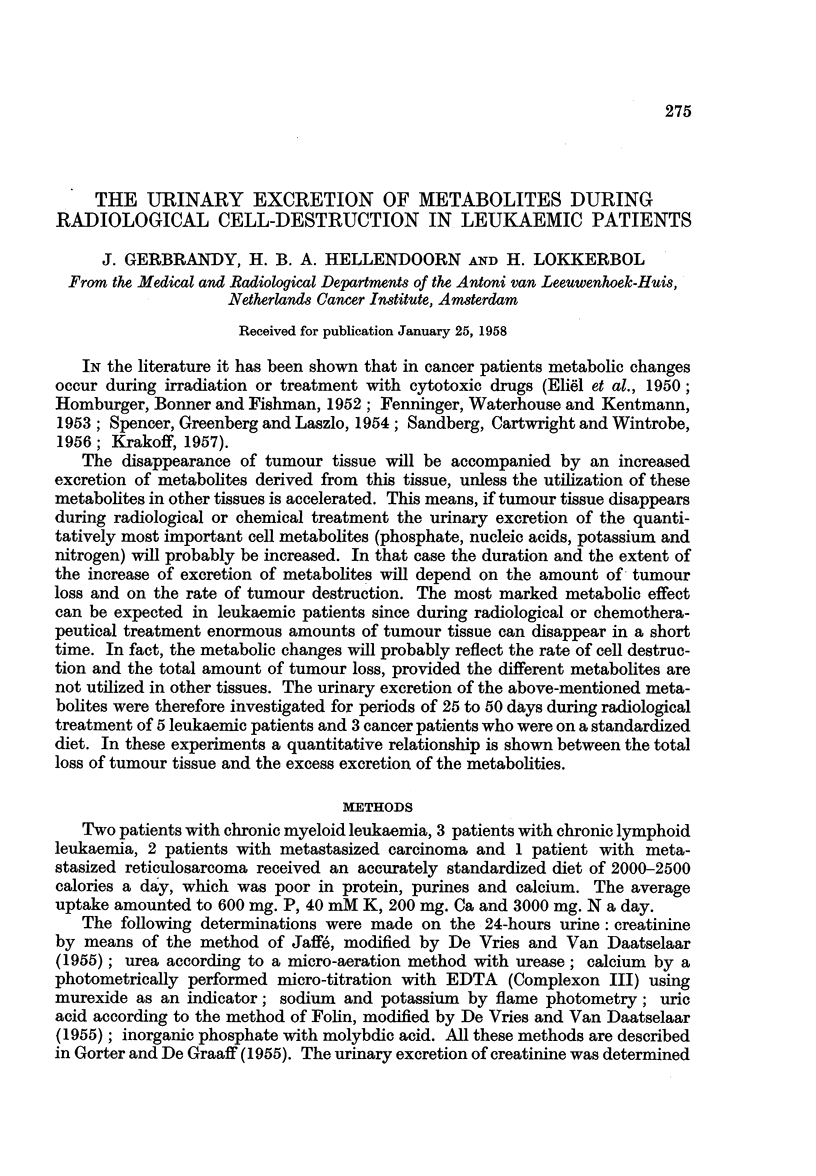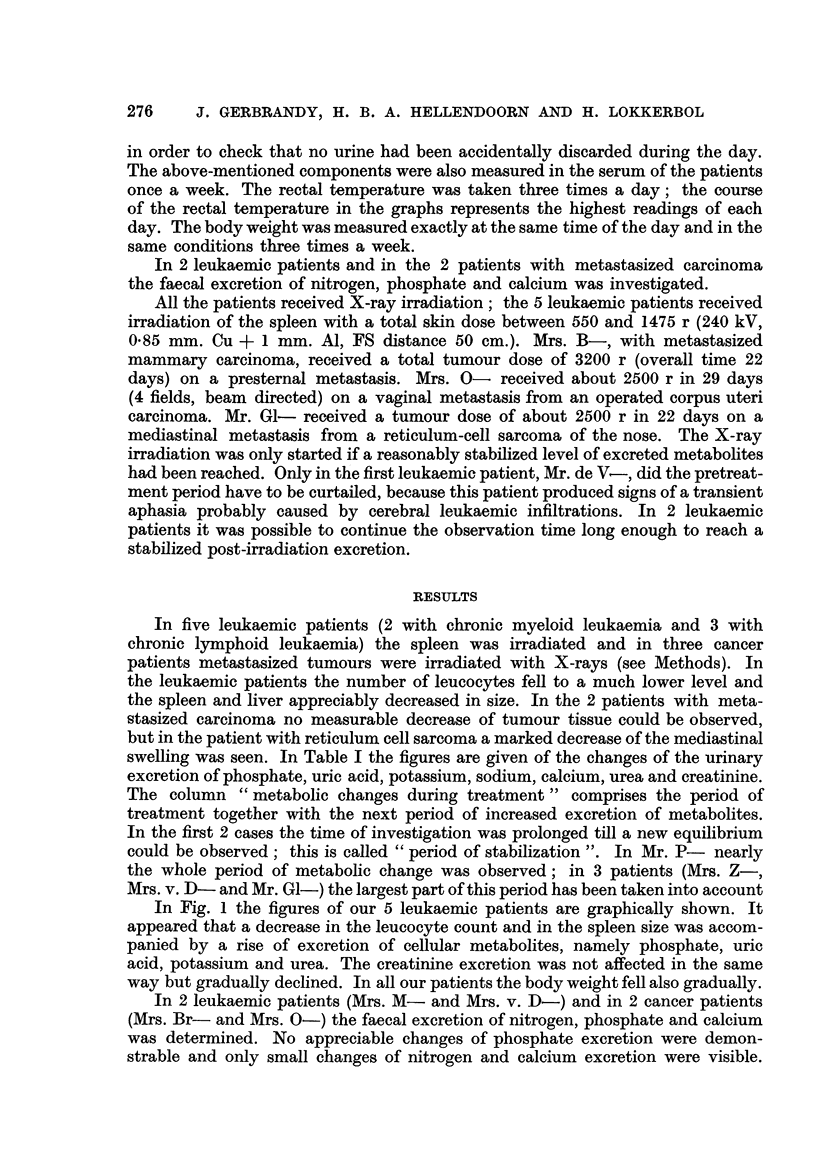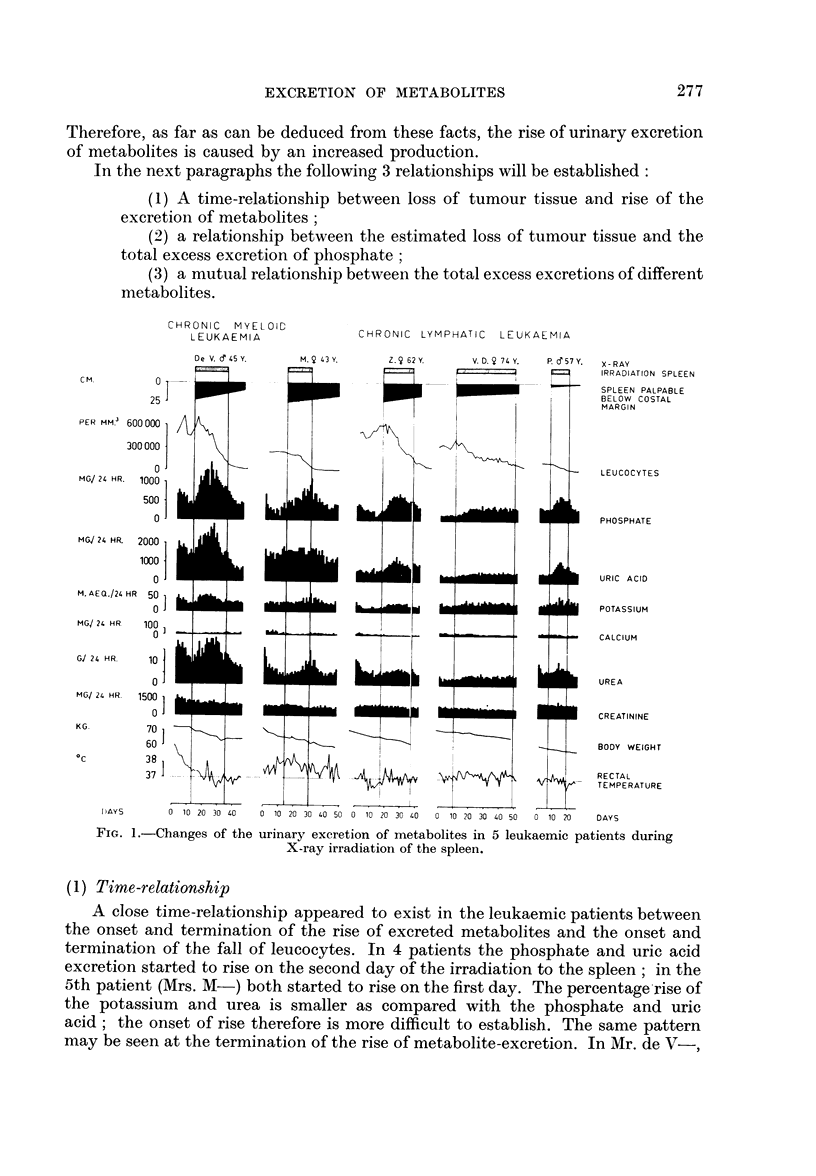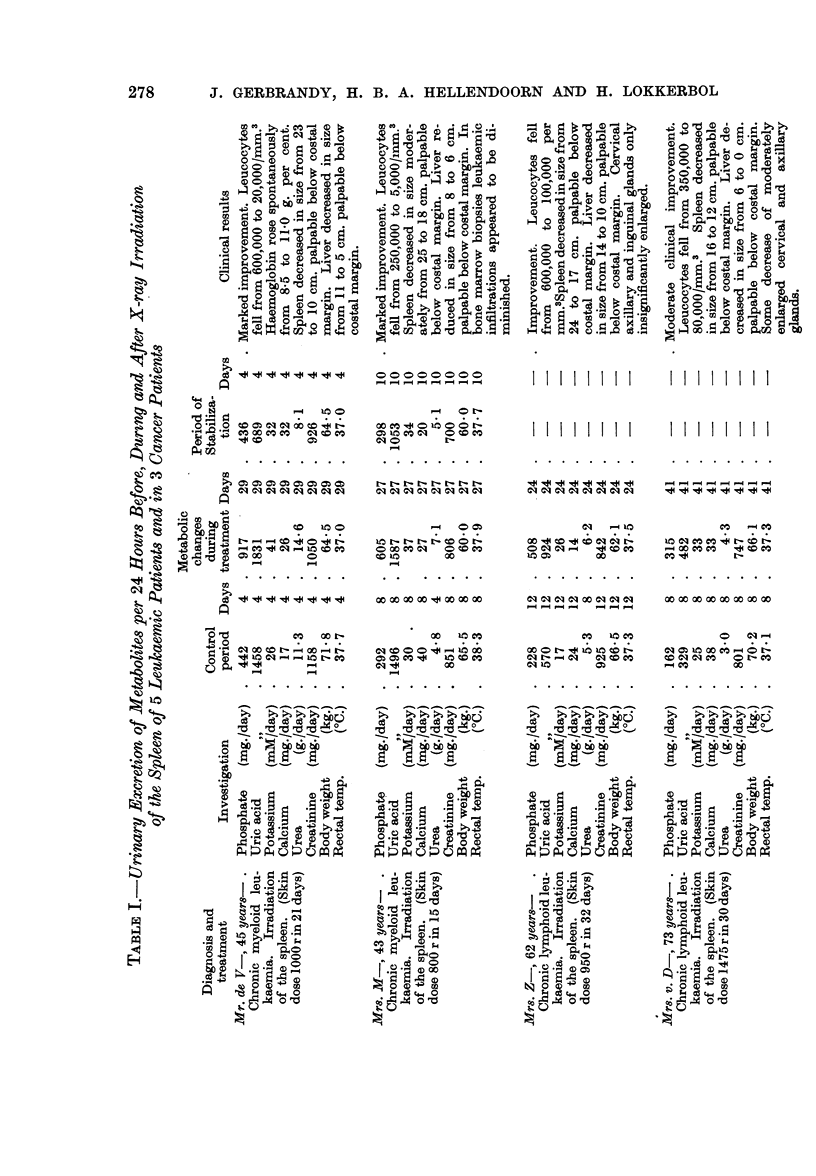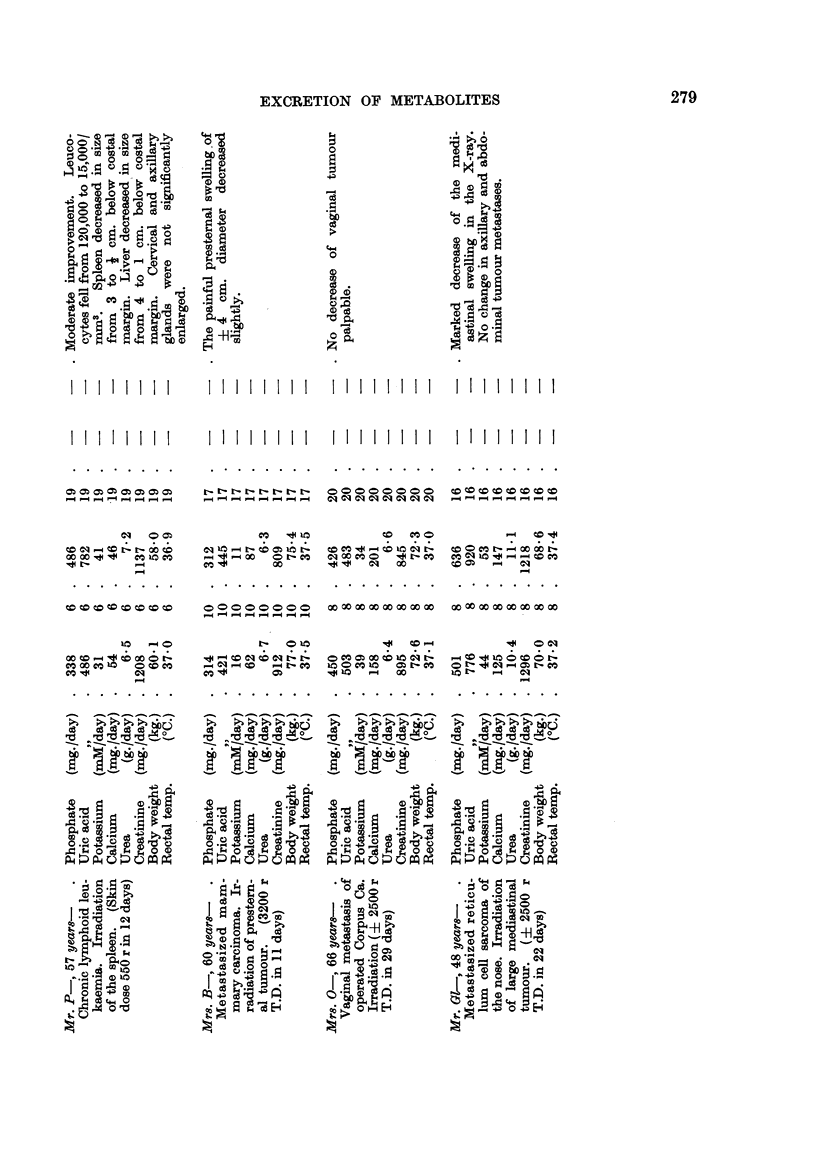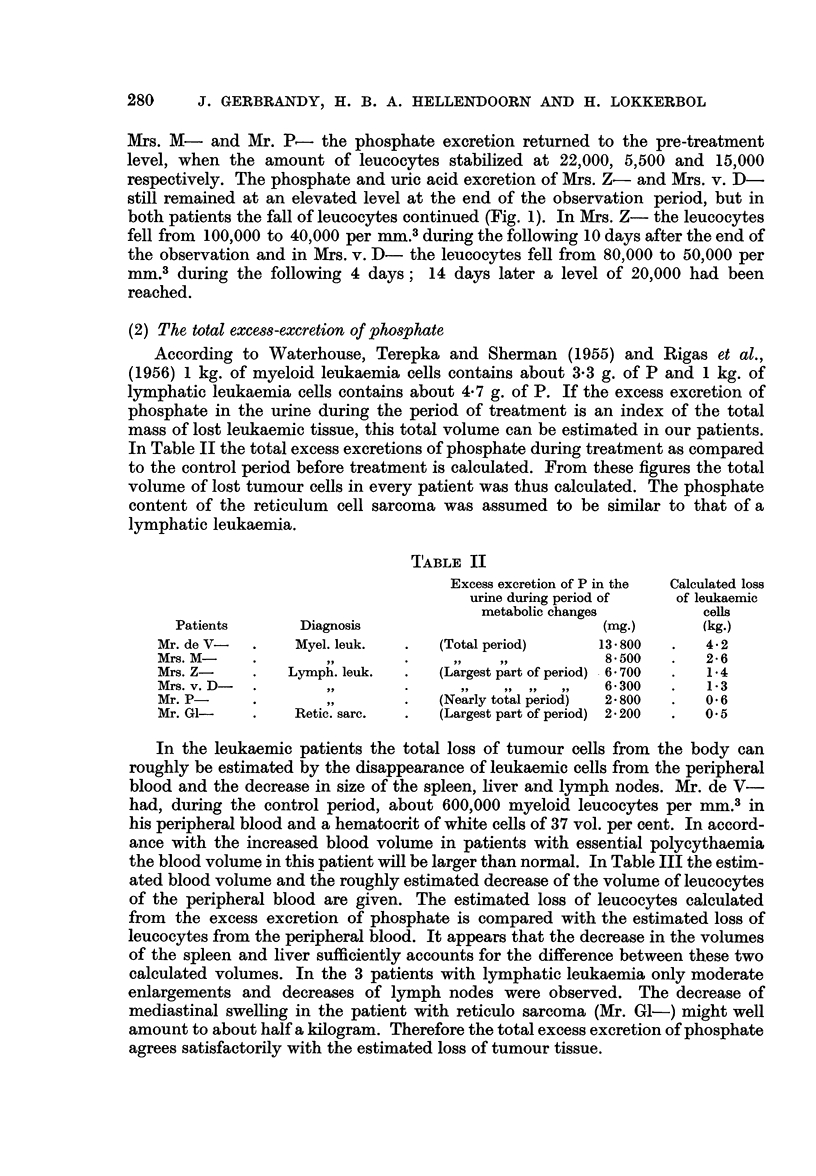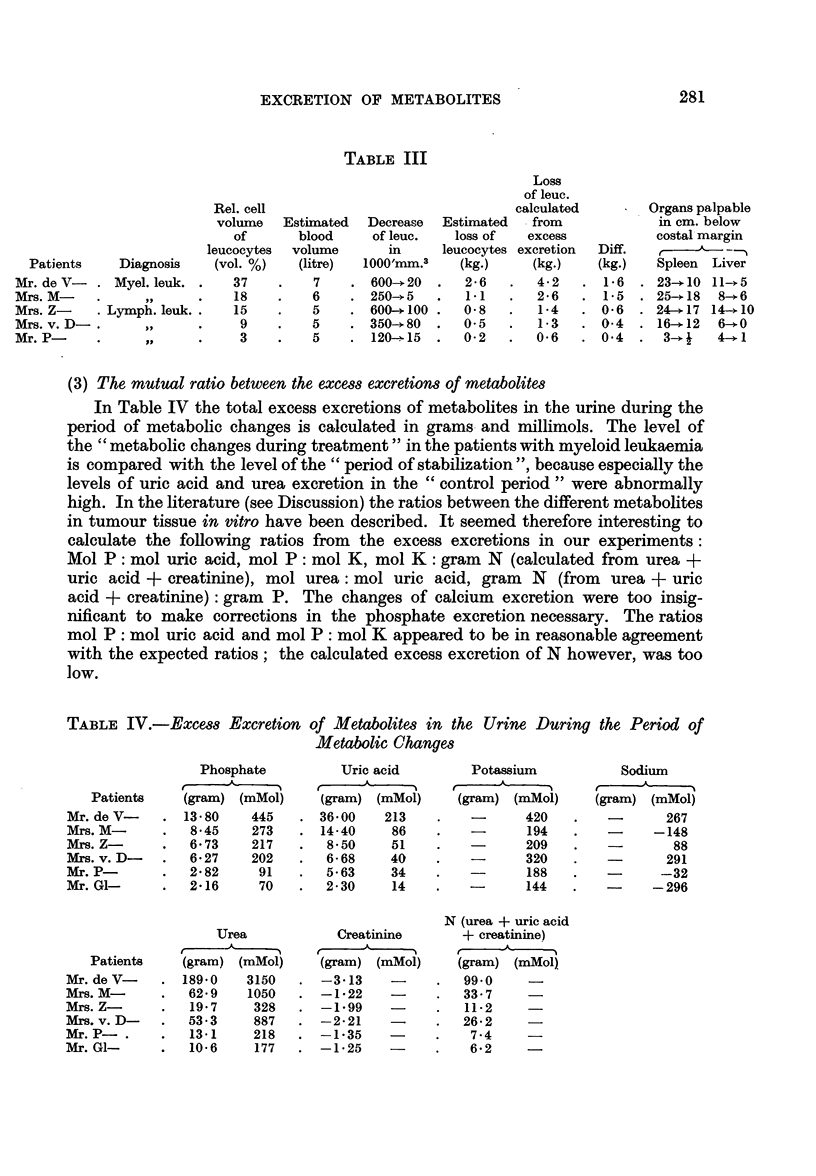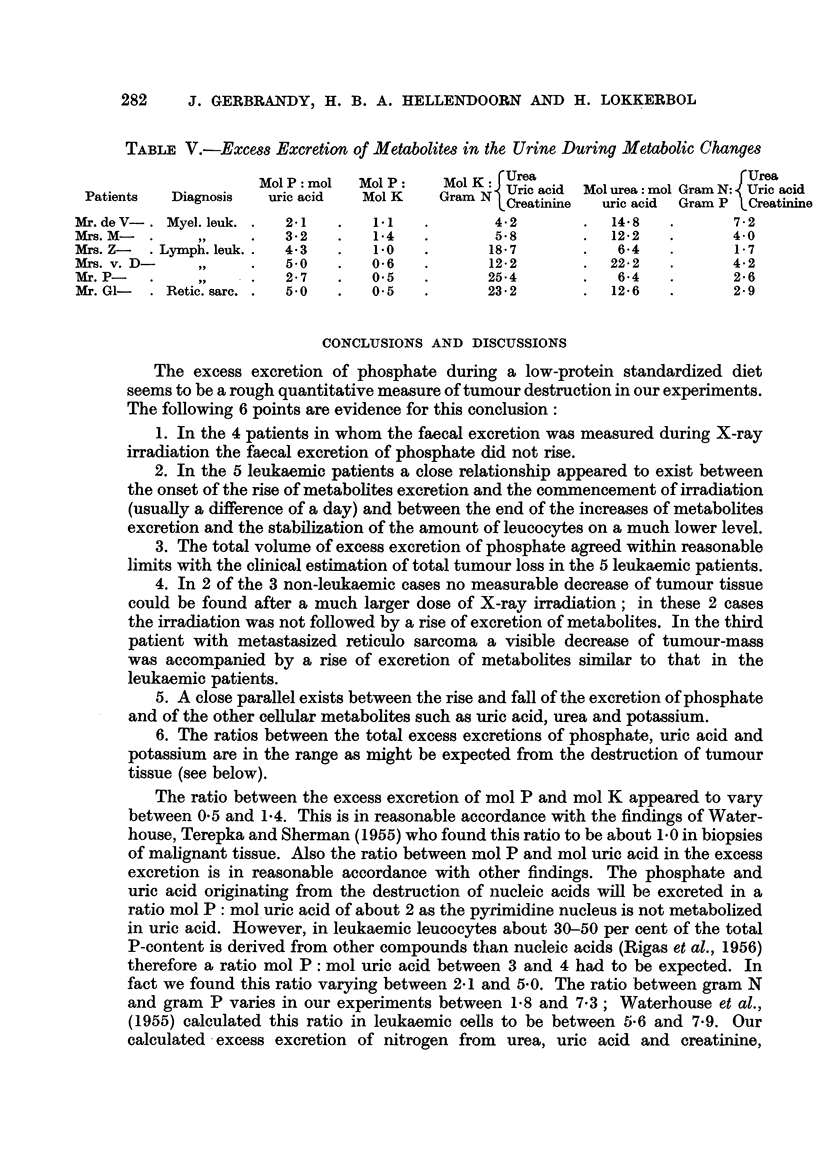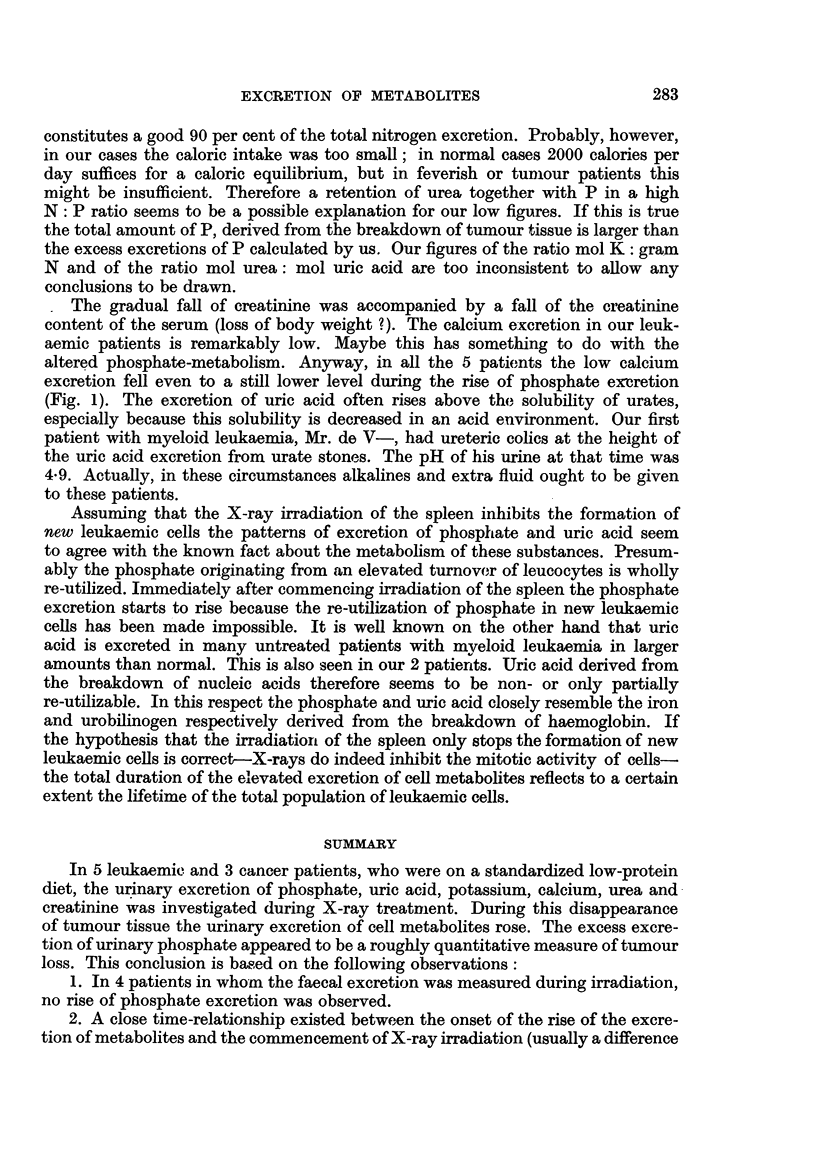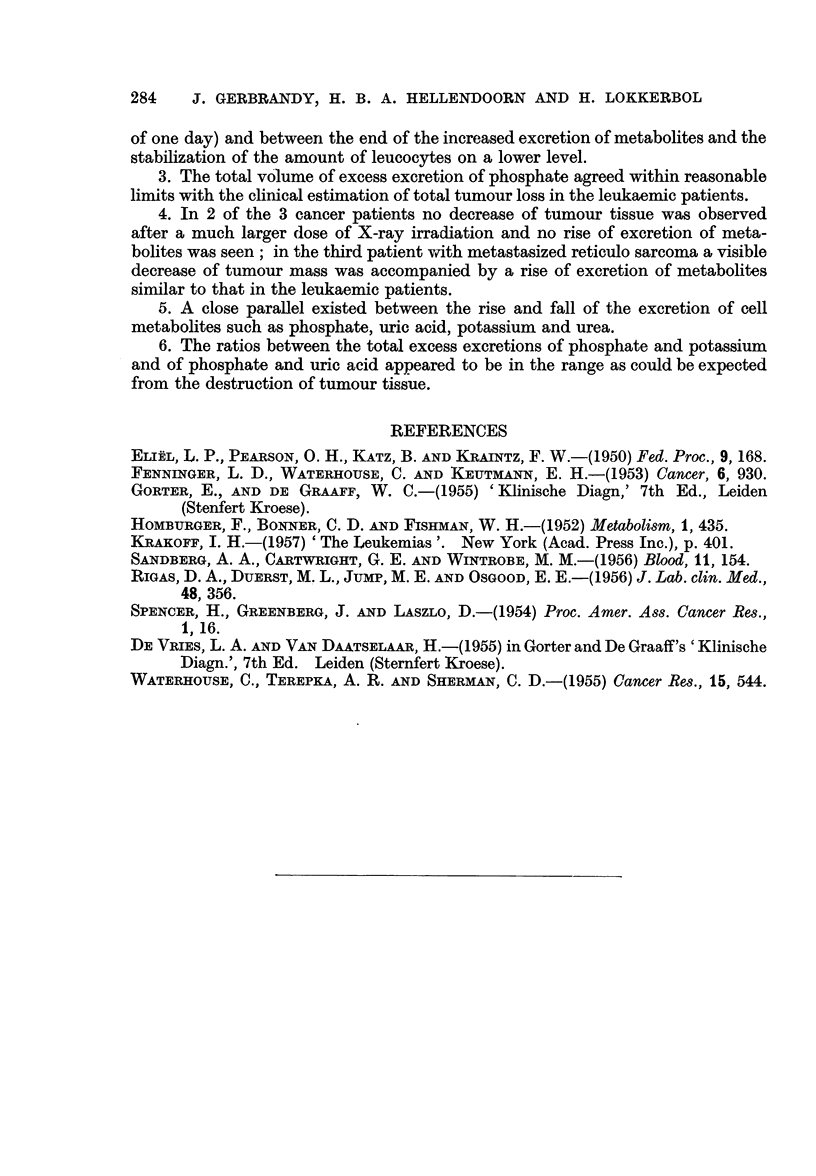# The Urinary Excretion of Metabolites During Radiological Cell-Destruction in Leukaemic Patients

**DOI:** 10.1038/bjc.1958.33

**Published:** 1958-06

**Authors:** J. Gerbrandy, H. B. A. Hellendoorn, H. Lokkerbol


### THE URINARY EXCRETION OF METABOLITES DURING RADIOLOGICAL CELL-DESTRUCTION IN LEUKAEMIC PATIENTS

J. GERBRANDY, H. B. A. HELLENDOORN AND H. LOKKERBOL

*From the Medical and Radiological Departments of the Antoni van Leeuwenhoek-Huis, Netherlands Cancer Institute, Amsterdam*

Received for publication January 25, 1958

IN the literature it has been shown that in cancer patients metabolic changes occur during irradiation or treatment with cytotoxic drugs (Eliël *et al.*, 1950; Homburger, Bonner and Fishman, 1952; Fenninger, Waterhouse and Kentmann, 1953; Spencer, Greenberg and Laszlo, 1954; Sandberg, Cartwright and Wintrobe, 1956; Krakoff, 1957).

The disappearance of tumour tissue will be accompanied by an increased excretion of metabolites derived from this tissue, unless the utilization of these metabolites in other tissues is accelerated. This means, if tumour tissue disappears during radiological or chemical treatment the urinary excretion of the quantitatively most important cell metabolites (phosphate, nucleic acids, potassium and nitrogen) will probably be increased. In that case the duration and the extent of the increase of excretion of metabolites will depend on the amount of tumour loss and on the rate of tumour destruction. The most marked metabolic effect can be expected in leukaemic patients since during radiological or chemotherapeutical treatment enormous amounts of tumour tissue can disappear in a short time. In fact, the metabolic changes will probably reflect the rate of cell destruction and the total amount of tumour loss, provided the different metabolites are not utilized in other tissues. The urinary excretion of the above-mentioned metabolites were therefore investigated for periods of 25 to 50 days during radiological treatment of 5 leukaemic patients and 3 cancer patients who were on a standardized diet. In these experiments a quantitative relationship is shown between the total loss of tumour tissue and the excess excretion of the metabolities.

### METHODS

Two patients with chronic myeloid leukaemia, 3 patients with chronic lymphoid leukaemia, 2 patients with metastasized carcinoma and 1 patient with metastasized reticulosarcoma received an accurately standardized diet of 2000–2500 calories a day, which was poor in protein, purines and calcium. The average uptake amounted to 600 mg. P, 40 mM K, 200 mg. Ca and 3000 mg. N a day.

The following determinations were made on the 24-hours urine: creatinine by means of the method of Jaffé, modified by De Vries and Van Daatselaar (1955); urea according to a micro-aeration method with urease; calcium by a photometrically performed micro-titration with EDTA (Complexon III) using murexide as an indicator; sodium and potassium by flame photometry; uric acid according to the method of Folin, modified by De Vries and Van Daatselaar (1955); inorganic phosphate with molybdic acid. All these methods are described in Gorter and De Graaff (1955). The urinary excretion of creatinine was determined in order to check that no urine had been accidentally discarded during the day. The above-mentioned components were also measured in the serum of the patients once a week. The rectal temperature was taken three times a day; the course of the rectal temperature in the graphs represents the highest readings of each day. The body weight was measured exactly at the same time of the day and in the same conditions three times a week.

In 2 leukaemic patients and in the 2 patients with metastasized carcinoma the faecal excretion of nitrogen, phosphate and calcium was investigated.

All the patients received X-ray irradiation; the 5 leukaemic patients received irradiation of the spleen with a total skin dose between 550 and 1475 r (240 kV, 0·85 mm. Cu + 1 mm. Al, FS distance 50 cm.). Mrs. B—, with metastasized mammary carcinoma, received a total tumour dose of 3200 r (overall time 22 days) on a presternal metastasis. Mrs. O— received about 2500 r in 29 days (4 fields, beam directed) on a vaginal metastasis from an operated corpus uteri carcinoma. Mr. Gl— received a tumour dose of about 2500 r in 22 days on a mediastinal metastasis from a reticulum-cell sarcoma of the nose. The X-ray irradiation was only started if a reasonably stabilized level of excreted metabolites had been reached. Only in the first leukaemic patient, Mr. de V—, did the pretreatment period have to be curtailed, because this patient produced signs of a transient aphasia probably caused by cerebral leukaemic infiltrations. In 2 leukaemic patients it was possible to continue the observation time long enough to reach a stabilized post-irradiation excretion.

### RESULTS

In five leukaemic patients (2 with chronic myeloid leukaemia and 3 with chronic lymphoid leukaemia) the spleen was irradiated and in three cancer patients metastasized tumours were irradiated with X-rays (see Methods). In the leukaemic patients the number of leucocytes fell to a much lower level and the spleen and liver appreciably decreased in size. In the 2 patients with metastasized carcinoma no measurable decrease of tumour tissue could be observed, but in the patient with reticulum cell sarcoma a marked decrease of the mediastinal swelling was seen. In Table I the figures are given of the changes of the urinary excretion of phosphate, uric acid, potassium, sodium, calcium, urea and creatinine. The column " metabolic changes during treatment " comprises the period of treatment together with the next period of increased excretion of metabolites. In the first 2 cases the time of investigation was prolonged till a new equilibrium could be observed; this is called " period of stabilization ". In Mr. P— nearly the whole period of metabolic change was observed; in 3 patients (Mrs. Z—, Mrs. v. D— and Mr. Gl—) the largest part of this period has been taken into account

In Fig. 1 the figures of our 5 leukaemic patients are graphically shown. It appeared that a decrease in the leucocyte count and in the spleen size was accompanied by a rise of excretion of cellular metabolites, namely phosphate, uric acid, potassium and urea. The creatinine excretion was not affected in the same way but gradually declined. In all our patients the body weight fell also gradually.

In 2 leukaemic patients (Mrs. M— and Mrs. v. D—) and in 2 cancer patients (Mrs. Br— and Mrs. O—) the faecal excretion of nitrogen, phosphate and calcium was determined. No appreciable changes of phosphate excretion were demonstrable and only small changes of nitrogen and calcium excretion were visible.

Therefore, as far as can be deduced from these facts, the rise of urinary excretion of metabolites is caused by an increased production.

In the next paragraphs the following 3 relationships will be established :

(1) A time-relationship between loss of tumour tissue and rise of the excretion of metabolites ;

(2) a relationship between the estimated loss of tumour tissue and the total excess excretion of phosphate ;

(3) a mutual relationship between the total excess excretions of different metabolites.

FIG. 1.—Changes of the urinary excretion of metabolites in 5 leukaemic patients during X-ray irradiation of the spleen.

### (1) *Time-relationship*

A close time-relationship appeared to exist in the leukaemic patients between the onset and termination of the rise of excreted metabolites and the onset and termination of the fall of leucocytes. In 4 patients the phosphate and uric acid excretion started to rise on the second day of the irradiation to the spleen ; in the 5th patient (Mrs. M—) both started to rise on the first day. The percentage rise of the potassium and urea is smaller as compared with the phosphate and uric acid ; the onset of rise therefore is more difficult to establish. The same pattern may be seen at the termination of the rise of metabolite-excretion. In Mr. de V—,

TABLE I.—*Urinary Excretion of Metabolites per 24 Hours Before, During and After X-ray Irradiation of the Spleen of 5 Leukaemic Patients and in 3 Cancer Patients*

| Diagnosis and treatment | Investigation | | Control period | Days | Metabolic changes during treatment | Days | Period of Stabilization | Days | Clinical results |
|---|---|---|---|---|---|---|---|---|---|
| *Mr. de V*—, 45 *years*— . Chronic myeloid leukaemia. Irradiation of the spleen. (Skin dose 1000 r in 21 days) | Phosphate | (mg./day) | 442 | 4 | 917 | 29 | 436 | 4 | Marked improvement. Leucocytes fell from 600,000 to 20,000/mm.³ Haemoglobin rose spontaneously from 8·5 to 11·0 g. per cent. Spleen decreased in size from 23 to 10 cm. palpable below costal margin. Liver decreased in size from 11 to 5 cm. palpable below costal margin. |
| | Uric acid | ,, | 1458 | 4 | 1831 | 29 | 689 | 4 | |
| | Potassium | (mM/day) | 26 | 4 | 41 | 29 | 32 | 4 | |
| | Calcium | (mg./day) | 17 | 4 | 26 | 29 | 32 | 4 | |
| | Urea | (g./day) | 11·3 | 4 | 14·6 | 29 | 8·1 | 4 | |
| | Creatinine | (mg./day) | 1158 | 4 | 1050 | 29 | 926 | 4 | |
| | Body weight | (kg.) | 71·8 | 4 | 64·5 | 29 | 64·5 | 4 | |
| | Rectal temp. | (°C.) | 37·7 | 4 | 37·0 | 29 | 37·0 | 4 | |
| *Mrs. M*—, 43 *years*— . Chronic myeloid leukaemia. Irradiation of the spleen. (Skin dose 800 r in 15 days) | Phosphate | (mg./day) | 292 | 8 | 605 | 27 | 298 | 10 | Marked improvement. Leucocytes fell from 250,000 to 5,000/mm.³ Spleen decreased in size moderately from 25 to 18 cm. palpable below costal margin. Liver reduced in size from 8 to 6 cm. palpable below costal margin. In bone marrow biopsies leukaemic infiltrations appeared to be diminished. |
| | Uric acid | ,, | 1496 | 8 | 1587 | 27 | 1053 | 10 | |
| | Potassium | (mM/day) | 30 | 8 | 37 | 27 | 34 | 10 | |
| | Calcium | (mg./day) | 40 | 8 | 27 | 27 | 20 | 10 | |
| | Urea | (g./day) | 4·8 | 4 | 7·1 | 27 | 5·1 | 10 | |
| | Creatinine | (mg./day) | 851 | 8 | 806 | 27 | 700 | 10 | |
| | Body weight | (kg.) | 65·5 | 8 | 60·0 | 27 | 60·0 | 10 | |
| | Rectal temp. | (°C.) | 38·3 | 8 | 37·9 | 27 | 37·7 | 10 | |
| *Mrs. Z*—, 62 *years*— . Chronic lymphoid leukaemia. Irradiation of the spleen. (Skin dose 950 r in 32 days) | Phosphate | (mg./day) | 228 | 12 | 508 | 24 | — | — | Improvement. Leucocytes fell from 600,000 to 100,000 per mm.³ Spleen decreased in size from 24 to 17 cm. palpable below costal margin. Liver decreased in size from 14 to 10 cm. palpable below costal margin. Cervical axillary and inguinal glands only insignificantly enlarged. |
| | Uric acid | ,, | 570 | 12 | 924 | 24 | — | — | |
| | Potassium | (mM/day) | 17 | 12 | 26 | 24 | — | — | |
| | Calcium | (mg./day) | 24 | 12 | 14 | 24 | — | — | |
| | Urea | (g./day) | 5·3 | 8 | 6·2 | 24 | — | — | |
| | Creatinine | (mg./day) | 925 | 12 | 842 | 24 | — | — | |
| | Body weight | (kg.) | 66·5 | 12 | 62·1 | 24 | — | — | |
| | Rectal temp. | (°C.) | 37·3 | 12 | 37·5 | 24 | — | — | |
| *Mrs. v. D*—, 73 *years*— . Chronic lymphoid leukaemia. Irradiation of the spleen. (Skin dose 1475 r in 30 days) | Phosphate | (mg./day) | 162 | 8 | 315 | 41 | — | — | Moderate clinical improvement. Leucocytes fell from 350,000 to 80,000/mm.³ Spleen decreased in size from 16 to 12 cm. palpable below costal margin. Liver decreased in size from 6 to 0 cm. palpable below costal margin. Some decrease of moderately enlarged cervical and axillary glands. |
| | Uric acid | ,, | 329 | 8 | 482 | 41 | — | — | |
| | Potassium | (mM/day) | 25 | 8 | 33 | 41 | — | — | |
| | Calcium | (mg./day) | 38 | 8 | 33 | 41 | — | — | |
| | Urea | (g./day) | 3·0 | 8 | 4·3 | 41 | — | — | |
| | Creatinine | (mg./day) | 801 | 8 | 747 | 41 | — | — | |
| | Body weight | (kg.) | 70·2 | 8 | 66·1 | 41 | — | — | |
| | Rectal temp. | (°C.) | 37·1 | 8 | 37·3 | 41 | — | — | |

| | | | | | | | | | |
|---|---|---|---|---|---|---|---|---|---|
| *Mr. P*—, 57 *years*—Chronic lymphoid leukaemia. Irradiation of the spleen. (Skin dose 550 r in 12 days) | Phosphate | (mg./day) | 338 | 6 | 486 | 19 | — | — | Moderate improvement. Leucocytes fell from 120,000 to 15,000/mm³. Spleen decreased in size from 3 to ½ cm. below costal margin. Liver decreased in size from 4 to 1 cm. below costal margin. Cervical and axillary glands were not significantly enlarged. |
| | Uric acid | ,, | 486 | 6 | 782 | 19 | — | — | |
| | Potassium | (mM/day) | 31 | 6 | 41 | 19 | — | — | |
| | Calcium | (mg./day) | 54 | 6 | 46 | 19 | — | — | |
| | Urea | (g./day) | 6·5 | 6 | 7·2 | 19 | — | — | |
| | Creatinine | (mg./day) | 1208 | 6 | 1137 | 19 | — | — | |
| | Body weight | (kg.) | 60·1 | 6 | 58·0 | 19 | — | — | |
| | Rectal temp. | (°C.) | 37·0 | 6 | 36·9 | 19 | — | — | |
| *Mrs. B*—, 60 *years*—Metastasized mammary carcinoma. Irradiation of presternal tumour. (3200 r T.D. in 11 days) | Phosphate | (mg./day) | 314 | 10 | 312 | 17 | — | — | The painful presternal swelling of ± 4 cm. diameter decreased slightly. |
| | Uric acid | ,, | 421 | 10 | 445 | 17 | — | — | |
| | Potassium | (mM/day) | 16 | 10 | 11 | 17 | — | — | |
| | Calcium | (mg./day) | 62 | 10 | 87 | 17 | — | — | |
| | Urea | (g./day) | 6·7 | 10 | 6·3 | 17 | — | — | |
| | Creatinine | (mg./day) | 912 | 10 | 809 | 17 | — | — | |
| | Body weight | (kg.) | 77·0 | 10 | 75·4 | 17 | — | — | |
| | Rectal temp. | (°C.) | 37·5 | 10 | 37·5 | 17 | — | — | |
| *Mrs. O*—, 66 *years*—Vaginal metastasis of operated Corpus Ca. Irradiation (± 2500 r T.D. in 29 days) | Phosphate | (mg./day) | 450 | 8 | 426 | 20 | — | — | No decrease of vaginal tumour palpable. |
| | Uric acid | ,, | 503 | 8 | 483 | 20 | — | — | |
| | Potassium | (mM/day) | 39 | 8 | 34 | 20 | — | — | |
| | Calcium | (mg./day) | 158 | 8 | 201 | 20 | — | — | |
| | Urea | (g./day) | 6·4 | 8 | 6·6 | 20 | — | — | |
| | Creatinine | (mg./day) | 895 | 8 | 845 | 20 | — | — | |
| | Body weight | (kg.) | 72·6 | 8 | 72·3 | 20 | — | — | |
| | Rectal temp. | (°C.) | 37·1 | 8 | 37·0 | 20 | — | — | |
| *Mr. Gl*—, 48 *years*—Metastasized reticulum cell sarcoma of the nose. Irradiation of large mediastinal tumour. (± 2500 r T.D. in 22 days) | Phosphate | (mg./day) | 501 | 8 | 636 | 16 | — | — | Marked decrease of the mediastinal swelling in the X-ray. No change in axillary and abdominal tumour metastases. |
| | Uric acid | ,, | 776 | 8 | 920 | 16 | — | — | |
| | Potassium | (mM/day) | 44 | 8 | 53 | 16 | — | — | |
| | Calcium | (mg./day) | 125 | 8 | 147 | 16 | — | — | |
| | Urea | (g./day) | 10·4 | 8 | 11·1 | 16 | — | — | |
| | Creatinine | (mg./day) | 1296 | 8 | 1218 | 16 | — | — | |
| | Body weight | (kg.) | 70·0 | 8 | 68·6 | 16 | — | — | |
| | Rectal temp. | (°C.) | 37·2 | 8 | 37·4 | 16 | — | — | |

Mrs. M— and Mr. P— the phosphate excretion returned to the pre-treatment level, when the amount of leucocytes stabilized at 22,000, 5,500 and 15,000 respectively. The phosphate and uric acid excretion of Mrs. Z— and Mrs. v. D— still remained at an elevated level at the end of the observation period, but in both patients the fall of leucocytes continued (Fig. 1). In Mrs. Z— the leucocytes fell from 100,000 to 40,000 per mm.$^3$ during the following 10 days after the end of the observation and in Mrs. v. D— the leucocytes fell from 80,000 to 50,000 per mm.$^3$ during the following 4 days; 14 days later a level of 20,000 had been reached.

(2) *The total excess-excretion of phosphate*

According to Waterhouse, Terepka and Sherman (1955) and Rigas *et al.*, (1956) 1 kg. of myeloid leukaemia cells contains about 3·3 g. of P and 1 kg. of lymphatic leukaemia cells contains about 4·7 g. of P. If the excess excretion of phosphate in the urine during the period of treatment is an index of the total mass of lost leukaemic tissue, this total volume can be estimated in our patients. In Table II the total excess excretions of phosphate during treatment as compared to the control period before treatment is calculated. From these figures the total volume of lost tumour cells in every patient was thus calculated. The phosphate content of the reticulum cell sarcoma was assumed to be similar to that of a lymphatic leukaemia.

TABLE II

| Patients | Diagnosis | Excess excretion of P in the urine during period of metabolic changes | (mg.) | Calculated loss of leukaemic cells (kg.) |
|---|---|---|---|---|
| Mr. de V— | Myel. leuk. | (Total period) | 13·800 | 4·2 |
| Mrs. M— | ,, | ,, ,, | 8·500 | 2·6 |
| Mrs. Z— | Lymph. leuk. | (Largest part of period) | 6·700 | 1·4 |
| Mrs. v. D— | ,, | ,, ,, ,, ,, | 6·300 | 1·3 |
| Mr. P— | ,, | (Nearly total period) | 2·800 | 0·6 |
| Mr. Gl— | Retic. sarc. | (Largest part of period) | 2·200 | 0·5 |

In the leukaemic patients the total loss of tumour cells from the body can roughly be estimated by the disappearance of leukaemic cells from the peripheral blood and the decrease in size of the spleen, liver and lymph nodes. Mr. de V— had, during the control period, about 600,000 myeloid leucocytes per mm.$^3$ in his peripheral blood and a hematocrit of white cells of 37 vol. per cent. In accordance with the increased blood volume in patients with essential polycythaemia the blood volume in this patient will be larger than normal. In Table III the estimated blood volume and the roughly estimated decrease of the volume of leucocytes of the peripheral blood are given. The estimated loss of leucocytes calculated from the excess excretion of phosphate is compared with the estimated loss of leucocytes from the peripheral blood. It appears that the decrease in the volumes of the spleen and liver sufficiently accounts for the difference between these two calculated volumes. In the 3 patients with lymphatic leukaemia only moderate enlargements and decreases of lymph nodes were observed. The decrease of mediastinal swelling in the patient with reticulo sarcoma (Mr. Gl—) might well amount to about half a kilogram. Therefore the total excess excretion of phosphate agrees satisfactorily with the estimated loss of tumour tissue.

TABLE III

| Patients | Diagnosis | Rel. cell volume of leucocytes (vol. %) | Estimated blood volume (litre) | Decrease of leuc. in 1000/mm.$^3$ | Estimated loss of leucocytes (kg.) | Loss of leuc. calculated from excess excretion (kg.) | Diff. (kg.) | Organs palpable in cm. below costal margin: Spleen | Liver |
|---|---|---|---|---|---|---|---|---|---|
| Mr. de V— | Myel. leuk. | 37 | 7 | 600→20 | 2·6 | 4·2 | 1·6 | 23→10 | 11→5 |
| Mrs. M— | „ | 18 | 6 | 250→5 | 1·1 | 2·6 | 1·5 | 25→18 | 8→6 |
| Mrs. Z— | Lymph. leuk. | 15 | 5 | 600→100 | 0·8 | 1·4 | 0·6 | 24→17 | 14→10 |
| Mrs. v. D— | „ | 9 | 5 | 350→80 | 0·5 | 1·3 | 0·4 | 16→12 | 6→0 |
| Mr. P— | „ | 3 | 5 | 120→15 | 0·2 | 0·6 | 0·4 | 3→½ | 4→1 |

(3) *The mutual ratio between the excess excretions of metabolites*

In Table IV the total excess excretions of metabolites in the urine during the period of metabolic changes is calculated in grams and millimols. The level of the "metabolic changes during treatment" in the patients with myeloid leukaemia is compared with the level of the "period of stabilization", because especially the levels of uric acid and urea excretion in the "control period" were abnormally high. In the literature (see Discussion) the ratios between the different metabolites in tumour tissue *in vitro* have been described. It seemed therefore interesting to calculate the following ratios from the excess excretions in our experiments: Mol P : mol uric acid, mol P : mol K, mol K : gram N (calculated from urea + uric acid + creatinine), mol urea : mol uric acid, gram N (from urea + uric acid + creatinine) : gram P. The changes of calcium excretion were too insignificant to make corrections in the phosphate excretion necessary. The ratios mol P : mol uric acid and mol P : mol K appeared to be in reasonable agreement with the expected ratios; the calculated excess excretion of N however, was too low.

TABLE IV.—*Excess Excretion of Metabolites in the Urine During the Period of Metabolic Changes*

| Patients | Phosphate (gram) | Phosphate (mMol) | Uric acid (gram) | Uric acid (mMol) | Potassium (gram) | Potassium (mMol) | Sodium (gram) | Sodium (mMol) |
|---|---|---|---|---|---|---|---|---|
| Mr. de V— | 13·80 | 445 | 36·00 | 213 | — | 420 | — | 267 |
| Mrs. M— | 8·45 | 273 | 14·40 | 86 | — | 194 | — | −148 |
| Mrs. Z— | 6·73 | 217 | 8·50 | 51 | — | 209 | — | 88 |
| Mrs. v. D— | 6·27 | 202 | 6·68 | 40 | — | 320 | — | 291 |
| Mr. P— | 2·82 | 91 | 5·63 | 34 | — | 188 | — | −32 |
| Mr. Gl— | 2·16 | 70 | 2·30 | 14 | — | 144 | — | −296 |

| Patients | Urea (gram) | Urea (mMol) | Creatinine (gram) | Creatinine (mMol) | N (urea + uric acid + creatinine) (gram) | N (urea + uric acid + creatinine) (mMol) |
|---|---|---|---|---|---|---|
| Mr. de V— | 189·0 | 3150 | −3·13 | — | 99·0 | — |
| Mrs. M— | 62·9 | 1050 | −1·22 | — | 33·7 | — |
| Mrs. Z— | 19·7 | 328 | −1·99 | — | 11·2 | — |
| Mrs. v. D— | 53·3 | 887 | −2·21 | — | 26·2 | — |
| Mr. P— . | 13·1 | 218 | −1·35 | — | 7·4 | — |
| Mr. Gl— | 10·6 | 177 | −1·25 | — | 6·2 | — |

TABLE V.—*Excess Excretion of Metabolites in the Urine During Metabolic Changes*

| Patients | Diagnosis | Mol P : mol uric acid | Mol P : Mol K | Mol K : Gram N (Urea, Uric acid, Creatinine) | Mol urea : mol uric acid | Gram N (Urea, Uric acid, Creatinine) : Gram P |
|---|---|---|---|---|---|---|
| Mr. de V— | Myel. leuk. | 2·1 | 1·1 | 4·2 | 14·8 | 7·2 |
| Mrs. M— | „ | 3·2 | 1·4 | 5·8 | 12·2 | 4·0 |
| Mrs. Z— | Lymph. leuk. | 4·3 | 1·0 | 18·7 | 6·4 | 1·7 |
| Mrs. v. D— | „ | 5·0 | 0·6 | 12·2 | 22·2 | 4·2 |
| Mr. P— | „ | 2·7 | 0·5 | 25·4 | 6·4 | 2·6 |
| Mr. Gl— | Retic. sarc. | 5·0 | 0·5 | 23·2 | 12·6 | 2·9 |

### CONCLUSIONS AND DISCUSSIONS

The excess excretion of phosphate during a low-protein standardized diet seems to be a rough quantitative measure of tumour destruction in our experiments. The following 6 points are evidence for this conclusion :

1. In the 4 patients in whom the faecal excretion was measured during X-ray irradiation the faecal excretion of phosphate did not rise.

2. In the 5 leukaemic patients a close relationship appeared to exist between the onset of the rise of metabolites excretion and the commencement of irradiation (usually a difference of a day) and between the end of the increases of metabolites excretion and the stabilization of the amount of leucocytes on a much lower level.

3. The total volume of excess excretion of phosphate agreed within reasonable limits with the clinical estimation of total tumour loss in the 5 leukaemic patients.

4. In 2 of the 3 non-leukaemic cases no measurable decrease of tumour tissue could be found after a much larger dose of X-ray irradiation ; in these 2 cases the irradiation was not followed by a rise of excretion of metabolites. In the third patient with metastasized reticulo sarcoma a visible decrease of tumour-mass was accompanied by a rise of excretion of metabolites similar to that in the leukaemic patients.

5. A close parallel exists between the rise and fall of the excretion of phosphate and of the other cellular metabolites such as uric acid, urea and potassium.

6. The ratios between the total excess excretions of phosphate, uric acid and potassium are in the range as might be expected from the destruction of tumour tissue (see below).

The ratio between the excess excretion of mol P and mol K appeared to vary between 0·5 and 1·4. This is in reasonable accordance with the findings of Waterhouse, Terepka and Sherman (1955) who found this ratio to be about 1·0 in biopsies of malignant tissue. Also the ratio between mol P and mol uric acid in the excess excretion is in reasonable accordance with other findings. The phosphate and uric acid originating from the destruction of nucleic acids will be excreted in a ratio mol P : mol uric acid of about 2 as the pyrimidine nucleus is not metabolized in uric acid. However, in leukaemic leucocytes about 30–50 per cent of the total P-content is derived from other compounds than nucleic acids (Rigas *et al.*, 1956) therefore a ratio mol P : mol uric acid between 3 and 4 had to be expected. In fact we found this ratio varying between 2·1 and 5·0. The ratio between gram N and gram P varies in our experiments between 1·8 and 7·3 ; Waterhouse *et al.*, (1955) calculated this ratio in leukaemic cells to be between 5·6 and 7·9. Our calculated excess excretion of nitrogen from urea, uric acid and creatinine, constitutes a good 90 per cent of the total nitrogen excretion. Probably, however, in our cases the caloric intake was too small; in normal cases 2000 calories per day suffices for a caloric equilibrium, but in feverish or tumour patients this might be insufficient. Therefore a retention of urea together with P in a high N : P ratio seems to be a possible explanation for our low figures. If this is true the total amount of P, derived from the breakdown of tumour tissue is larger than the excess excretions of P calculated by us. Our figures of the ratio mol K : gram N and of the ratio mol urea : mol uric acid are too inconsistent to allow any conclusions to be drawn.

The gradual fall of creatinine was accompanied by a fall of the creatinine content of the serum (loss of body weight ?). The calcium excretion in our leukaemic patients is remarkably low. Maybe this has something to do with the altered phosphate-metabolism. Anyway, in all the 5 patients the low calcium excretion fell even to a still lower level during the rise of phosphate excretion (Fig. 1). The excretion of uric acid often rises above the solubility of urates, especially because this solubility is decreased in an acid environment. Our first patient with myeloid leukaemia, Mr. de V—, had ureteric colics at the height of the uric acid excretion from urate stones. The pH of his urine at that time was 4·9. Actually, in these circumstances alkalines and extra fluid ought to be given to these patients.

Assuming that the X-ray irradiation of the spleen inhibits the formation of *new* leukaemic cells the patterns of excretion of phosphate and uric acid seem to agree with the known fact about the metabolism of these substances. Presumably the phosphate originating from an elevated turnover of leucocytes is wholly re-utilized. Immediately after commencing irradiation of the spleen the phosphate excretion starts to rise because the re-utilization of phosphate in new leukaemic cells has been made impossible. It is well known on the other hand that uric acid is excreted in many untreated patients with myeloid leukaemia in larger amounts than normal. This is also seen in our 2 patients. Uric acid derived from the breakdown of nucleic acids therefore seems to be non- or only partially re-utilizable. In this respect the phosphate and uric acid closely resemble the iron and urobilinogen respectively derived from the breakdown of haemoglobin. If the hypothesis that the irradiation of the spleen only stops the formation of new leukaemic cells is correct—X-rays do indeed inhibit the mitotic activity of cells—the total duration of the elevated excretion of cell metabolites reflects to a certain extent the lifetime of the total population of leukaemic cells.

### SUMMARY

In 5 leukaemic and 3 cancer patients, who were on a standardized low-protein diet, the urinary excretion of phosphate, uric acid, potassium, calcium, urea and creatinine was investigated during X-ray treatment. During this disappearance of tumour tissue the urinary excretion of cell metabolites rose. The excess excretion of urinary phosphate appeared to be a roughly quantitative measure of tumour loss. This conclusion is based on the following observations :

1. In 4 patients in whom the faecal excretion was measured during irradiation, no rise of phosphate excretion was observed.

2. A close time-relationship existed between the onset of the rise of the excretion of metabolites and the commencement of X-ray irradiation (usually a difference of one day) and between the end of the increased excretion of metabolites and the stabilization of the amount of leucocytes on a lower level.

3. The total volume of excess excretion of phosphate agreed within reasonable limits with the clinical estimation of total tumour loss in the leukaemic patients.

4. In 2 of the 3 cancer patients no decrease of tumour tissue was observed after a much larger dose of X-ray irradiation and no rise of excretion of metabolites was seen ; in the third patient with metastasized reticulo sarcoma a visible decrease of tumour mass was accompanied by a rise of excretion of metabolites similar to that in the leukaemic patients.

5. A close parallel existed between the rise and fall of the excretion of cell metabolites such as phosphate, uric acid, potassium and urea.

6. The ratios between the total excess excretions of phosphate and potassium and of phosphate and uric acid appeared to be in the range as could be expected from the destruction of tumour tissue.

### REFERENCES

Eliël, L. P., Pearson, O. H., Katz, B. and Kraintz, F. W.—(1950) *Fed. Proc.*, **9**, 168.

Fenninger, L. D., Waterhouse, C. and Keutmann, E. H.—(1953) *Cancer*, **6**, 930.

Gorter, E., and de Graaff, W. C.—(1955) 'Klinische Diagn,' 7th Ed., Leiden (Stenfert Kroese).

Homburger, F., Bonner, C. D. and Fishman, W. H.—(1952) *Metabolism*, **1**, 435.

Krakoff, I. H.—(1957) 'The Leukemias'. New York (Acad. Press Inc.), p. 401.

Sandberg, A. A., Cartwright, G. E. and Wintrobe, M. M.—(1956) *Blood*, **11**, 154.

Rigas, D. A., Duerst, M. L., Jump, M. E. and Osgood, E. E.—(1956) *J. Lab. clin. Med.*, **48**, 356.

Spencer, H., Greenberg, J. and Laszlo, D.—(1954) *Proc. Amer. Ass. Cancer Res.*, **1**, 16.

De Vries, L. A. and Van Daatselaar, H.—(1955) in Gorter and De Graaff's 'Klinische Diagn.', 7th Ed. Leiden (Sternfert Kroese).

Waterhouse, C., Terepka, A. R. and Sherman, C. D.—(1955) *Cancer Res.*, **15**, 544.